# Warming Increases the Spread of an Invasive Thistle

**DOI:** 10.1371/journal.pone.0021725

**Published:** 2011-06-29

**Authors:** Rui Zhang, Eelke Jongejans, Katriona Shea

**Affiliations:** 1 Department of Biology, The Pennsylvania State University, University Park, Pennsylvania, United States of America; 2 Department of Experimental Plant Ecology, Institute for Water and Wetland Research, Radboud University Nijmegen, Nijmegen, The Netherlands; University of Tartu, Estonia

## Abstract

**Background:**

Global warming and shifted precipitation regimes increasingly affect species abundances and distributions worldwide. Despite a large literature on species' physiological, phenological, growth, and reproductive responses to such climate change, dispersal is rarely examined. Our study aims to test whether the dispersal ability of a non-native, wind-dispersed plant species is affected by climate change, and to quantify the ramifications for future invasion spread rates.

**Methodology/Principal Findings:**

We experimentally increased temperature and precipitation in a two-cohort, factorial field study (n = 80). We found an overwhelming warming effect on plant life history: warming not only improved emergence, survival, and reproduction of the thistle *Carduus nutans*, but also elevated plant height, which increased seed dispersal distances. Using spatial population models, we demonstrate that these empirical warming effects on demographic vital rates, and dispersal parameters, greatly exacerbate spatial spread. Predicted levels of elevated winter precipitation decreased seed production per capitulum, but this only slightly offset the warming effect on spread. Using a spread rate decomposition technique (c*-LTRE), we also found that plant height-mediated changes in dispersal contribute most to increased spread rate under climate change.

**Conclusions/Significance:**

We found that both dispersal and spread of this wind-dispersed plant species were strongly impacted by climate change. Dispersal responses to climate change can improve, or diminish, a species' ability to track climate change spatially, and should not be overlooked. Methods that combine both demographic and dispersal responses thus will be an invaluable complement to projections of suitable habitat under climate change.

## Introduction

In the face of climate change, dispersal imposes a major limit on a species' ability to keep pace with environmental shifts [1], [2], and thus determines the fate of individuals, population persistence, and species distributions [3], [4]. Species with low dispersal ability are more likely to suffer from range contraction and eventual extinction, particularly in fragmented habitats [5]. This is especially true for species with limited environmental tolerances and when evolutionary responses occur more slowly than climate change [6]. Invasive species, on the other hand, are often associated with high dispersal and may become more problematic under changing climate [7].

Although the importance of including dispersal in climate change studies has been raised repeatedly [8], dispersal is rarely examined in this context [9]. The lack of dispersal data often leads to unrealistic assumptions of unlimited dispersal, or no dispersal at all, when predicting future species distributions [8]. The disparate predictions resulting from these two extreme cases [10], [11] underline the necessity of including explicit dispersal information to reduce the significant uncertainties caused by spread when projecting species distribution shifts. Furthermore, even when dispersal under current climate is known, extrapolation of present dispersal abilities to the future remains a challenge. Factors affecting dispersal processes, such as the architectural features of maternal plants, the morphology of dispersal units [12], and dispersal vectors [*7*] may not stay constant over environmental gradients; thus, dispersal may also be altered by climate change. Therefore it is necessary to evaluate possible changes in dispersal when examining species' responses to climate change. However, no study so far has examined dispersal-related plant responses to future climate scenarios. Neither does any study couple altered dispersal processes with altered population dynamics under climate change to assess future population performance, although theoretical approaches exist to reach this goal [13], [14], [15].

Here we present an experimental and theoretical study which addresses dispersal responses in addition to responses of survival, growth, and reproduction of a wind-dispersed invasive thistle, *Carduus nutans*, to climate change. Dispersal, although recognized as a critical component in invasion processes [16], [17], is generally overlooked when examining responses of invasive species under climate change. We divide the responses of *C. nutans* into two categories: i). responses in demographic vital rates, such as seedling emergence, rosette survival, bolting rate, capitulum production, seed production per capitulum; ii). responses that relate to dispersal characteristics, such as plant height at flowering (which affects seed release height), and propagule terminal velocity (which depends on seed weight and pappus morphology).

## Results

We examined two independent cohorts in a former pasture in central Pennsylvania. We manipulated both temperature and precipitation. In half of our plots, fiberglass open top chambers (OTCs) passively increased daily average temperature on the soil surface by 0.58°C across seasons in the two years of experiment (corresponding to an annual increase of 97 degree days). We also achieved a 30% increase in winter precipitation with or without a 15% increase in summer precipitation by manually adding rain or snow – these percentages are the extremes from regional climate projections for this area [18], [19]. Other vegetation was suppressed in all plots to mimic the disturbed environments (e.g. overgrazed pastures) in which this species attains maximum invasion success.

Although our increase in temperature was mild compared to regional projections (increases of 2.9°C–5.3°C in annual surface air temperature by the end of this century [19]), we found significant responses of *C. nutans* to warming. Seedling emergence in the fall was enhanced in warmed plots (25±3% versus 19±3%, mean±s.e., n = 80, generalized linear mixed-effects model (GLMM) with quasibinomial error structure to account for overdispersion, n = 80, *P*<0.001). Warming also increased rosette overwinter survival (95±2% versus 87±3%, binomial GLMM, n = 80, *P* = 0.0085). Bolting probability was not different between warmed and ambient plots for plants that survived the winter (86±4% versus 90±3%, binomial GLMM, n = 79, *P* = 0.70). Plant reproduction was significantly increased by warming. Both mature flower head production (49.84±5.60 versus 36.65±4.08, GLMM based on log linear transformed data, n = 77, *P* = 0.0028) and total (including buds) capitulum production (76.04±9.62 versus 57.41±6.75, GLMM based on log linear transformed data, n = 77, *P* = 0.028) were higher in warmed plots. Seed number per capitulum was not affected by warming (522±29 versus 476±31, GLMM, n = 77, *P* = 0.19). Precipitation addition in both winter and summer had no significant effect on any of the above vital rates. Increased precipitation in winter alone only reduced seed number per capitulum (427±50 versus 539±28, GLMM, n = 77, *P* = 0.0080).

We combined the percentage increases in seedling emergence (+29.53%), survival (+9.35%), and total capitulum production (+32.44%) due to warming, and decreases in seed number per capitulum due to increased winter precipitation (−20.79%) with a baseline 4×4 demographic matrix model (based on vital rates for an invasion experiment population at the same site from a previous study [14], see also Text S1, Table S1, Table S2) to calculate the long-term population growth rate λ under three climate change scenarios: warming, winter precipitation addition, and warming with winter precipitation addition. We assumed that the proportional responses to warming are size-independent, the establishment rate of emerging seedlings is not changed under warming, and all buds counted at harvest would have continued to set seed. We projected an 87% increase in per capita population growth rate under warming (680 versus 363), a 20% decrease under winter precipitation addition alone (288 versus 362), and a 49% increase under both warming and increased winter precipitation (539 versus 362).

**Figure 1:**
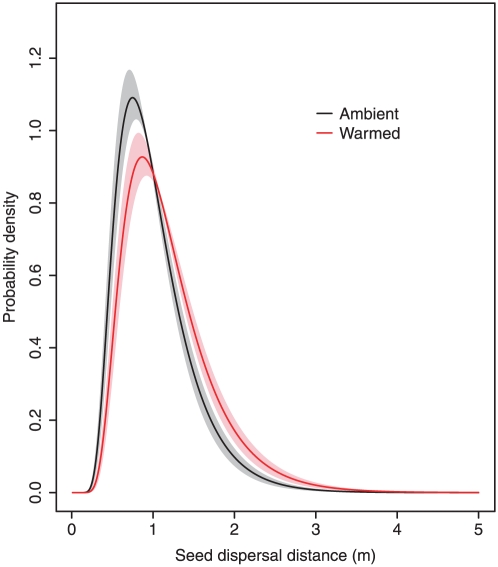
Seed dispersal kernels for *C. nutans* grown in ambient and warmed conditions. The bands (grey for ambient; pink for warmed) illustrate the standard deviations based on bootstrapped plant height from the experiment, while the lines represent the mean of the bootstrapped curves (see Text S1).

In our study, plant height, a dispersal-related trait, was enhanced in warmed plots by 11.88 cm, or 9% (151.75±4.94 cm versus 139.87±4.23 cm, GLMM, n = 77, *P* = 0.0033). Warming did not have any significant effect on seed terminal velocity (0.75±0.04 m/s (ambient) versus 0.74 ± 0.04 m/s (warmed), GLMM, n = 66, *P* = 0.80), seed weight (3.42±0.09 mg versus 3.36±0.12 mg, GLMM, n = 66, *P* = 0.80), or pappus diameter (21.67±1.0 mm versus 21.58±1.0 mm, GLMM, n = 66, *P* = 0.81). Increased precipitation did not have any significant effect on seed weight (n = 66, GLMM, *P* = 0.90): 3.38±0.09 mg (ambient), 3.38±0.16 mg (winter precipitation addition), 3.46±0.18 mg (winter and summer precipitation addition). The effects of precipitation addition on pappus diameter and terminal velocity were not consistent for the two cohorts (Table S1). Therefore the only consistent and significant effects of climate change on dispersal are mediated via the enhanced plant height at flowering. We calculated dispersal kernels for both ambient and warmed conditions using the Wald analytical long-distance dispersal (WALD) model [20], [21]. We parameterized the model with terminal velocities and plant flowering height (assumed to be the same as seed release height) from our experiment, while horizontal wind velocity data from a nearby weather station collected during the main dispersal months were used to estimate mean wind speed and a turbulence parameter [14]. Increased plant flowering height under warming led to a distinct dispersal kernel compared to the control (Figure 1); each seed has a higher probability of travelling farther under warmed than under ambient conditions.

**Figure 2:**
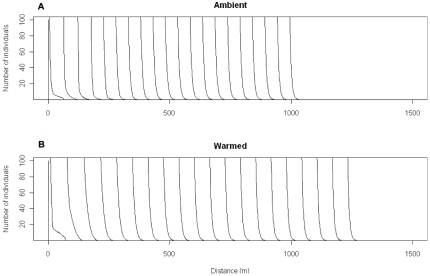
Visualizations of invasion waves in 20 years based on two random simulations. A) Ambient condition. B) Warmed condition. Note that the front of the wave converged to a constant speed and shape after a few years.

**Figure 3:**
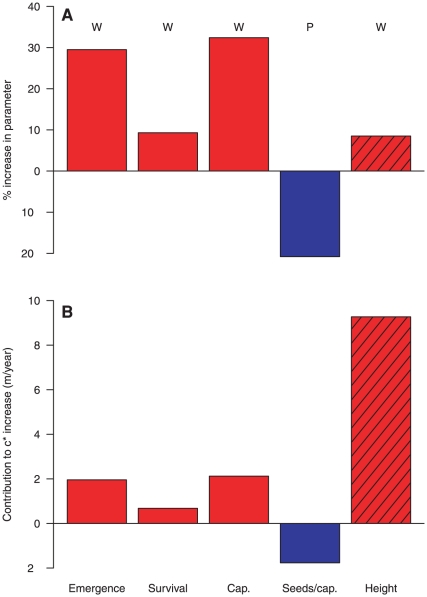
Changes in demographic and dispersal-related traits and their contributions to increased spread rate. A) Mean percentage changes in demographic and dispersal- related traits. B) Their corresponding contributions to increased spread rate. Warming induced changes in fall seedling emergence, survival, capitulum production, and flowering plant height: W and in red. Mean percentage change in seed number per capitulum due to increased winter precipitation: P and in blue. Solid: demographic vital rates. Hatched: dispersal-related traits.

Spatial population spread results from a combination of 1) local demographic processes (which determine how many propagules are produced) and 2) the dispersal process itself (which determines how far each propagule moves). We coupled demographic models with dispersal models to project the population spread rate, c*, using integrodifference equations [13]–[15]. We used baseline data from an earlier invasion study [14] and modified the models based on our experimental results (Table S1, Table S2). The population spread rate increased by 27% under warming (66 m/year versus 52 m/year, the medians of 100,000 simulations under increased or ambient temperatures, see simulations in Figure 2). In other words, it takes about 19 years for the invasion front to move 1 km under ambient conditions, while only 15 years under warming. Increased winter precipitation alone, which affected seed production per capitulum, did not have a large impact on the spread rate (50 m/year versus 52 m/year). Increased winter precipitation and warming together led to a 23% increase in the spread rate (64 m/year versus 52 m/year). This implies that warming (with or without precipitation increases) is likely to cause more rapid spread of this invasive species once it establishes, even in the absence of other (e.g. human-mediated) dispersal pathways. We used a c*- LTRE [14] approach to decompose the increase in c* under warming with increased winter precipitation into contributions by the parameter differences. Increased height made a much larger contribution to increased spread rate than did the demographic vital rates (Figure 3); warming-induced changes in plant height may have the strongest influence on population spread of *C. nutans* under climate change.

## Discussion

As shown by our results, climate change affects many aspects of species life histories. While enhanced emergence, survival and reproduction directly contribute to local population dynamics [22], altered dispersal characteristics can affect the ability of offspring to arrive in suitable habitats and to buffer against harsh environments spatially. Here warming enhances the spread rate of an invasive wind-dispersed species through both demographic and dispersal processes, with enhanced dispersal contributing disproportionately to increased spread. This is because the invasion wave speed is very sensitive to plant height [14]. While we parameterized our model with data from an experimental population where growing conditions were optimal for invasion (i.e. little competition and abundant resources), we also assessed the spread rate using data from a naturally established population in Kansas. Though its contribution diminishes compared to the increased contribution of emergence (probably due to strong competition in natural populations), increased dispersal still plays an important role in increased spread under warming (Figure S1).

Our results suggest that the invasion of this species can be exacerbated by climate change, resulting largely from enhanced dispersal because of elevated plant height. Previous studies have documented that many invasive species are able to track local climate change, via adjusted phenology [23], improved establishment [24], and enhanced biomass and reproduction [25]–[28]. Our study demonstrates that some invasive species can also benefit from enhanced dispersal-related traits, which may increase their chance of reaching in newly suitable habitats. Furthermore, although possible changes in dispersal processes accompanied with climate change have been raised in recent reviews [7], [16], these studies consider extrinsic factors, such as altered transportation and deliberate introduction, rather than the traits of the plant itself. Our results, however, illustrate the possibility of altered plant dispersal traits due to climate change, which can interact with extrinsic factors involved in dispersal processes to produce synergistic or antagonistic outcomes.

Our experiment was conducted to maximize the invasion response of this species. In particular, plants were grown with very little competition from surrounding vegetation, thus mimicking scenarios in frequently disturbed areas, such as overgrazed pastures. Spread rates in such habitats are projected to increase under climate change, and our results suggest that management efforts should be focused more on the control of dispersal relative to demographic processes. While our study focuses on the maximal responses of the species, future studies should also address other biotic factors, such as the responses of surrounding vegetation to climate change and thereby possibly modified competition, and interference with dispersal. As well as biotic responses, future research based on this approach may also include environmental factors that affect dispersal processes, such as changes in wind speed [29] and turbulence [30] under future climate scenarios. For example, a recently published study by Nathan et al. [31] combined demographic and dispersal processes with projected wind speed changes to project wind-driven spread of tree species under climate change. Such approaches will complement biotic envelope models, most of which do not address biotic processes such as population dynamics and dispersal [32]. Evaluations of species dispersal and spread abilities, and changes in these abilities, under climate change will provide important insights for both conservation of endangered species, which often lack efficient dispersal, and management of invasive species.

## Materials and Methods

### Study species

*Carduus nutans* L. (Asteraceae) is an introduced Eurasian weed, which causes major economic problems in many regions in the world [15]. *C. nutans* is a monocarpic, short-lived (winter annual in our experiment) thistle and produces a large number of wind-dispersed seeds [21].

### Field site descriptions

We conducted our experiments at The Russell E. Larson Agricultural Research Farm at Rock Springs, Pennsylvania, USA (latitude 40.71°, longitude −77.94°). The field site was a former pasture, which was dominated by *Arrhenatherum elatius*, *Dactylis glomerata*, *Elytrigia repens*, *Phleum pratense*, *Taraxacum* spp., *Plantago lanceolata*, *Linaria vulgaris, Trifolium* spp., and *Galium* spp. The field site was disked and all aboveground vegetation was removed prior to planting each cohort, to mimic the high invasion success of this opportunistic species in disturbed habitats.

### Treatments

Our climate manipulation incorporated both elevated temperature and increased precipitation according to the regional climate projections in the Northeastern U.S. Open top chambers (OTCs) were used to elevate temperature in the experiment. These OTCs were 0.4 m in height and 1.5 m in basal diameter, and were constructed according to the International Tundra Experiment Manual [33]. Cumulative degree-days for *C. nutans*, calculated based on McCarty [34], were significantly higher in plots with OTCs, with the largest increase in each spring (81.40±11.65 degree-days). OTCs did not have a significant effect on soil moisture or snow depth based on field measurements. Therefore we assume that elevated temperature was the predominant abiotic factor altered by the OTCs. We manipulated both winter (December - February) and summer (June–July) precipitation. Passive precipitation collectors were used to collect natural precipitation in the field. Rain and snow were manually added after each precipitation event.

### Experimental design

In each cohort, we set up ten blocks, each containing four plots (one control, one with warming, one with added precipitation, and one with both treatments). As most climate projections agree with increasing future winter precipitation in U.S. Northeast, whereas summer projections are highly variable [35], precipitation plots in half of the blocks received winter precipitation addition only (+30%), while precipitation plots in the other blocks also received summer addition (+15%). In each fall, we transplanted four three-week-old rosettes into 2 m×2 m plots, either with or without OTCs. Field censuses were conducted weekly in the following growing season. Other plot vegetation was kept short by hand clipping. We terminated our experiments in the following late July by harvesting all aboveground biomass. Capitula were separated and counted based on developmental stage. One mature flower head per plot was used to examine seed production per capitulum, seed traits, and terminal velocities (i.e. the speed at which a propagule eventually falls in still air when drag equals the force of gravity; faster drops lead to shorter travel distances), following the methods of Marchetto et al. [36]. Fall seedling emergence was examined by sowing twenty-five seeds into the center of each plot in the October of 2008 and 2009. Emerged seedlings were recorded and killed every two days until December.

### Statistics

Analyses based on plot averages were performed in R [37] using mixed linear models (lmer) with appropriate error structure specifications or transformations of the responses. Shapiro-Wilks tests were used to verify normality. Full models started with fixed effects of warming, precipitation and their interaction (except for seedling emergence where precipitation was not relevant), and the random effects of cohort and block. Initial rosette size at transplanting was a covariate (except for seedling emergence). Stepwise model simplifications were based on lower AIC values. Bolting probability was analyzed as the conditional probability given rosettes survived the winter. Responses of reproduction and height were averaged for flowering plants in each plot before the analyses. The arithmetic means of seed weight and pappus diameter, as well as the geometric mean of terminal velocity of seeds within each flower head were used when analyzing effects of climate change on seed dispersal.

### Models

We used intergrodifference equations to couple matrix models of population dynamics with a WALD model of dispersal to calculate spread rates (Text S1). We used the c*- LTRE technique to decompose the increase in spread rate into contributions of model parameter changes as in [14].

## Supporting Information

Figure S1 **Modelling results based on a natural population.** A) Mean percentage changes in fall seedling emergence, survival, capitulum production, and flowering plant height due to warming (W, in red) and mean percentage change in seed number per capitulum due to both warming and increased winter precipitation (WP, in purple). B) Their corresponding contributions to increased spread rate. Solid: demographic vital rates. Hatched: dispersal-related traits. The spread rate was 21 m/year for ambient treatments and 32 m/year for warmed treatments with increased winter precipitation. Spread rates were calculated based on a natural population in Kansas [3] using the c*-LTRE technique. The calculated percentage change in reproduction (+4.9%) combines both increased capitulum production and decreased seed production per capitulum, as the original data only contained the product of these two terms: seed production per flowering plants [9].
(EPS)

Table S1 **Summary of the main results of the study.**
(DOC)

Table S2 **Vital rates and dispersal-related parameters used in the demographic models and spread models.**
(DOC)

Text S1 **Supporting Information.**
(DOC)
