# Supplementary material for: Warming Increases the Spread of an Invasive Thistle
Source: PLoS One. 2011 Jun 29;6(6):e21725. doi: 10.1371/journal.pone.0021725 (PMC3126854; doi:10.1371/journal.pone.0021725)
Supplement: Table S1 — Summary of the main results of the study. (DOC) [file pone.0021725.s002.doc]

**Table S1** Summary of the main results of the study

|  |  | Temperature | | Winter precipitation | |
| --- | --- | --- | --- | --- | --- |
|  | Responses* | Ambient | Warmed | Ambient | Increased by 30% |
| Demography | Fall emergence (%) | 19^a^ | 25^b^ | / | / |
|  | Winter survival (%) | 87^a^ | 95^b^ | 92^c^ | 90^c^ |
|  | Bolting probability (%) | 90^a^ | 86^a^ | 88^c^ | 84^c^ |
|  | Total capitulum production | 57^a^ | 76^b^ | 66^c^ | 57^c^ |
|  | Seeds per capitulum | 476^a^ | 522^a^ | 539^c^ | 427^d^ |
| Dispersal | Height at flowering (cm) | 140^a^ | 152^b^ | 145^c^ | 145^c^ |
|  | Pappus diameter (mm)† | 21.7^a^ | 21.6^a^ | 18.1^c^ (cohort 1)  26.8^c^ (cohort 2) | 20.5^c^ (cohort 1)  20.7^d^ (cohort 2) |
|  | Seed weight (mg) | 3.42^a^ | 3.36^a^ | 3.38^c^ | 3.38^c^ |
|  | Terminal velocity (m/s) ‡ | 0.75^a^ | 0.74^a^ | 0.90^c^ (cohort 1)  0.54^c^ (cohort 2) | 0.81^c^ (cohort 1)  0.77^d^ (cohort 2) |

*The superscripts a and b denote the statistical significance between ambient temperature and warmed treatments, and the superscripts c and d denote the statistical significance between ambient precipitation and increased winter precipitation treatments. No significant interaction was found between temperature and winter precipitation manipulation.

†The effect of increased winter precipitation on pappus diameter is not consistent between the two cohorts. No significant effect was found for the first cohort (18.09 ± 1.00 mm (ambient), 20.49 ± 2.17 mm (winter precipitation addition), 16.90 ± 1.53 mm (winter and summer precipitation addition), GLMM, n = 32, *P*>0.05). In the second cohort, precipitation addition only in winter led to a significantly shorter pappus diameter, while precipitation in both winter and summer was not different from control (26.83 ± 0.93 mm (ambient), 20.74 ± 1.99 mm (winter precipitation addition), 24.37 ± 1.27 mm (winter and summer precipitation addition), GLMM, n = 32, *P*<0.001 for the comparison of winter precipitation addition versus ambient, *P*=0.24 for the comparison of both winter and summer precipitation addition versus ambient).

‡The effect of increased precipitation on seed terminal velocity is not consistent for the two cohorts. No significant effect was found for the first cohort (0.90 ± 0.06 m/s (ambient), 0.80 ± 0.08 m/s (winter precipitation addition), 0.85 ± 0.07 m/s (winter and summer precipitation addition), GLMM, based on log transformation, n = 32, *P*>0.05), whereas in the second cohort terminal velocities were significantly higher (i.e. seeds drop more quickly) for treatments with increased winter precipitation than in the ambient condition (0.54 ± 0.03 m/s (ambient), 0.77 ± 0.07 m/s (winter precipitation addition), 0.66 ± 0.03 m/s (winter and summer precipitation addition), GLMM, based on log transformation, n = 34 , *P*<0.05). As this result is inconsistent for the two cohorts, we ignore the effect of increased winter precipitation on seed terminal velocity in the spread rate modeling.
