# Supplementary material for: Warming Increases the Spread of an Invasive Thistle
Source: PLoS One. 2011 Jun 29;6(6):e21725. doi: 10.1371/journal.pone.0021725 (PMC3126854; doi:10.1371/journal.pone.0021725)
Supplement: Table S2 — Vital rates and dispersal-related parameters used in the demographic models and spread models. (DOC) [file pone.0021725.s003.doc]

**Table S2** Vital rates and dispersal-related parameters used in the demographic models and spread models

|  |  | Experimental population* | | | | Natural population† | | | |
| --- | --- | --- | --- | --- | --- | --- | --- | --- | --- |
|  |  | ambient | warm | water | warm+water | ambient | warm | water | warm+water |
| σ_1_ | survival Survival of seed in seed bank | 0.2597 | 0.2597 | 0.2597 | 0.2597 | 0.2597 | 0.2597 | 0.2597 | 0.2597 |
| **σ_2_** | **Survival of small rosettes (S)** | **0.2619** | **0.2864** | **0.2619** | **0.2864** | **0.2182** | **0.2386** | **0.2182** | **0.2386** |
| **σ_3_** | **Survival of median rosettes (M)** | **0.6761** | **0.7393** | **0.6761** | **0.7393** | **0.5633** | **0.6160** | **0.5633** | **0.6160** |
| **σ_4_** | **Survival of large rosettes (L)** | **0.8971** | **0.9810** | **0.8971** | **0.9810** | **0.7474** | **0.8172** | **0.7474** | **0.8172** |
| γ_3_ | Growth of establishing seed to M | 0.2076 | 0.2076 | 0.2076 | 0.2076 | 0.1627 | 0.1627 | 0.1627 | 0.1627 |
| γ_4_ | Growth of establishing seed to L | 0.0911 | 0.0911 | 0.0911 | 0.0911 | 0.0000 | 0.0000 | 0.0000 | 0.0000 |
| γ_32_ | Growth of surviving, not-bolting S to M | 0.8028 | 0.8028 | 0.8028 | 0.8028 | 0.8028 | 0.8028 | 0.8028 | 0.8028 |
| γ_42_ | Growth of surviving, not-bolting S to L | 0.1268 | 0.1268 | 0.1268 | 0.1268 | 0.1268 | 0.1268 | 0.1268 | 0.1268 |
| γ_43_ | Growth of surviving, not-bolting M to L | 0.3824 | 0.3824 | 0.3824 | 0.3824 | 0.3824 | 0.3824 | 0.3824 | 0.3824 |
| ρ_23_ | Retrogression of surviving, not bolting M to S | 0.0000 | 0.0000 | 0.0000 | 0.0000 | 0.0000 | 0.0000 | 0.0000 | 0.0000 |
| ρ_24_ | Retrogression of surviving, not bolting L to S | 0.0000 | 0.0000 | 0.0000 | 0.0000 | 0.0000 | 0.0000 | 0.0000 | 0.0000 |
| ρ_34_ | Retrogression of surviving, not bolting L to M | 0.0000 | 0.0000 | 0.0000 | 0.0000 | 0.0000 | 0.0000 | 0.0000 | 0.0000 |
| β_2_ | Bolting of surviving S | 0.1932 | 0.1932 | 0.1932 | 0.1932 | 0.0180 | 0.0180 | 0.0180 | 0.0180 |
| β_3_ | Bolting of surviving M | 0.7143 | 0.7143 | 0.7143 | 0.7143 | 0.3596 | 0.3596 | 0.3596 | 0.3596 |
| β_4_ | Bolting of surviving L | 1.0000 | 1.0000 | 1.0000 | 1.0000 | 1.0000 | 1.0000 | 1.0000 | 1.0000 |
| **π_2_** | **Potential seed production by S** | **5443** | **7809** | **4311** | **5710** | **100** | **132** | **79** | **105** |
| **π_3_** | **Potential seed production by M** | **6150** | **8145** | **4871** | **6451** | **195** | **258** | **154** | **205** |
| **π_4_** | **Potential seed production by L** | **12446** | **16483** | **9858** | **13056** | **1473** | **1951** | **1167** | **1545** |
| φ | Potential seed escaping from floral herbivory | 0.8500 | 0.8500 | 0.8500 | 0.8500 | 1.0000 | 1.0000 | 1.0000 | 1.0000 |
| ν | New seed entering seed bank | 0.2333 | 0.2333 | 0.2333 | 0.2333 | 0.2333 | 0.2333 | 0.2333 | 0.2333 |
| **ε** | **New seed establishing seedling** | **0.2333** | **0.3022** | **0.2333** | **0.3022** | **0.0300** | **0.3022** | **0.2333** | **0.3022** |
| **ε_1_** | **Seed from seed bank establishing seedling** | **0.2333** | **0.3022** | **0.2333** | **0.3022** | **0.0300** | **0.3022** | **0.2333** | **0.3022** |
| **F** | **Seed terminal velocity** | **0.7107** | **0.7107** | **0.7107** | **0.7107** | **0.7107** | **0.7107** | **0.7107** | **0.7107** |
| **σ_F_** | **Standard deviation of ln F** | **0.3003** | **0.3003** | **0.3003** | **0.3003** | **0.3003** | **0.3003** | **0.3003** | **0.3003** |
| **H** | **Plant height (m)** | **140** | **152** | **140** | **152** | **140** | **152** | **140** | **152** |
| h | Vegetation height (m) | 50 | 50 | 50 | 50 | 30 | 30 | 30 | 30 |
| U | Wind speed (m s^-1^) at 10m | 1.7257 | 1.7257 | 1.7257 | 1.7257 | 2.7332 | 2.7332 | 2.7332 | 2.7332 |
| σ_U_ | Standard deviation of ln U | 0.7288 | 0.7288 | 0.7288 | 0.7288 | 0.6522 | 0.6522 | 0.6522 | 0.6522 |
|  |  |  |  |  |  |  |  |  |  |
| **λ** | **Projected population growth rate** | **363** | **680** | **288** | **539** | **1.748** | **2.430** | **1.581** | **2.181** |
| **c*** | **Projected spread rate (m year^-1^)** | **52** | **66** | **50** | **64** | **21** | **36** | **18** | **32** |

Modifications on parameter estimates based on the present study are in bold. Others were assumed to be the same as in previous studies. Warm: plots that were warmed using OTCs. Water: plots that received a 30% increase in winter precipitation. Warm + Water: plots that received both warming treatment and winter precipitation addition.

* The estimations for the experimental population were based on a previous experiment conducted in Pennsylvania (K. Shea unpublished data 2002-2005), where competition and floral herbivory were suppressed by regularly weeding and application of insecticide after bolting. Please refer to Jongejans et al. [3] for more details about the experiment.

† The estimations for the natural population were based on a naturally occurring population in Kansas which has been described in Lee and Hamrick [9].
