# Supplementary material for: Warming Increases the Spread of an Invasive Thistle
Source: PLoS One. 2011 Jun 29;6(6):e21725. doi: 10.1371/journal.pone.0021725 (PMC3126854; doi:10.1371/journal.pone.0021725)
Supplement: Text S1 — Supporting Information. (DOC) [file pone.0021725.s004.doc]

**Supporting Information**

**Demographic model**

The demographic models were based on existing demographic data for this species (e.g. [1], [2]), modified according to the proportional changes of vital rates based on our experimental results. We used vital rate-based matrix models for *C. nutans* as presented by Jongejans et al. [3], based on a four-stage classification (which represent annual demographic transitions from the end of autumn of one year to the end of autumn a year later):

Seed bank (SB) Small (S) Medium (M) Large (L) (1)

$$\left(\begin{matrix} \sigma_{1} & \sigma_{2}\beta_{2}\pi_{2}\phi\nu& \sigma_{3}\beta_{3}\pi_{3}\phi\nu& \sigma_{4}\beta_{4}\pi_{4}\phi\nu\\ \varepsilon_{1}(1-\gamma_{3}-\gamma_{4}) & \sigma_{2}\text{(1-}\beta_{2}\text{)}(1-\gamma_{32}-\gamma_{42})\text{+}\sigma_{2}\beta_{2}\pi_{2}\phi\varepsilon(1-\gamma_{3}-\gamma_{4}) & \sigma_{3}\text{(1-}\beta_{3}\text{)}\rho_{23}\text{+}\sigma_{3}\beta_{3}\pi_{3}\phi\varepsilon(1-\gamma_{3}-\gamma_{4}) & \sigma_{4}\text{(1-}\beta_{4}\text{)}\rho_{24}\text{+}\sigma_{4}\beta_{4}\pi_{4}\phi\varepsilon(1-\gamma_{3}-\gamma_{4}) \\ \varepsilon_{1}\gamma_{3} & \sigma_{2}\text{(1-}\beta_{2}\text{)}\gamma_{32}\text{+}\sigma_{2}\beta_{2}\pi_{2}\phi\varepsilon\gamma_{3} & \sigma_{3}\text{(1-}\beta_{3}\text{)}(1-\rho_{23}-\gamma_{43})\text{+}\sigma_{3}\beta_{3}\pi_{3}\phi\varepsilon\gamma_{3} & \sigma_{4}\text{(1-}\beta_{4}\text{)}\rho_{34}\text{+}\sigma_{4}\beta_{4}\pi_{4}\phi\varepsilon\gamma_{3} \\ \varepsilon_{1}\gamma_{4} & \sigma_{2}\text{(1-}\beta_{2}\text{)}\gamma_{42}\text{+}\sigma_{2}\beta_{2}\pi_{2}\phi\varepsilon\gamma_{4} & \sigma_{3}\text{(1-}\beta_{3}\text{)}\gamma_{43}\text{+}\sigma_{3}\beta_{3}\pi_{3}\phi\varepsilon\gamma_{4} & \sigma_{4}\text{(1-}\beta_{4}\text{)}(1-\rho_{24}-\rho_{34})\text{+}\sigma_{4}\beta_{4}\pi_{4}\phi\varepsilon\gamma_{4} \end{matrix}\right)$$

We then incorporated into the models significant proportional changes in seedling emergence (ε, ε_1_), survival (σ_2_, σ_3_, σ_4_), and reproduction (π_2_, π_3_, π_4_) caused by climate change, to project population growth rate *λ*. Bolting probabilities (β_2_, β_3_, β_4_), transitions between stages due to growth (γ_3_, γ_4_, γ_32_, γ_42_, γ_34_), potential seed escaping from floral herbivory (φ), and new seed entering the seed bank (ν) were assumed to remain the same.
Dispersal model

We modelled dispersal using the mechanistic Wald Analytical Long-distance Dispersal (WALD) model [4], which we had shown applies well for C. nutans in a major field experiment [5]. This model was derived from well-established models in fluid mechanics and predicts an inverse Gaussian (Wald) distribution of dispersal distances r [6]:

$p\left(r\right)=\left(\frac{\xi'}{2\pi r^{3}}\right)^{\frac{1}{2}}\exp\left(-\frac{\xi'\left(r-\mu'\right)^{2}}{2\mu'^{2}r}\right)$ (2)

where location parameter *μ*’ and the scale parameter *ξ*’ are given by:

$\mu'=\frac{HU}{F}$ and $\xi'=\left(\frac{H}{\sigma}\right)^{2}$ (3)

where *H* is the seed release height (mean plant height), *F* is seed terminal settling velocity, *U* is the hourly mean horizontal wind velocity between *H* and the ground, and *σ* is a turbulent flow parameter which reflects wind variation due to vegetation structure and weather conditions [4]. We used H and F calculated based on the present experiment, as well as *U* and *σ* estimated based on weather data collected in a nearby weather station, to parameterize the dispersal model.

We bootstrapped the plant height data 1000 times, while keeping the other parameters unchanged, and calculated the corresponding dispersal kernels. We then calculated the mean and standard deviation of the bootstrapping results, which were presented in Fig 1.

Spread model

The demographic and dispersal models were combined to allow the modeling of spatial spread using integrodifference equations [2, 3, 7]:

H = MA (4)

where A is the demographic model, and signifies the Hadamard product (element by element multiplication), and M is a matrix of the same size as A, which is comprised of elements containing the moment generating function of the dispersal kernel k(r) (eqn 6). For population growth rates less than unity and for thin-tailed (exponentially bounded) dispersal kernels (e.g. the WALD model), the population spread rate converges to a constant speed, *c**:

$c*=\min_{j>0} \frac{1}{j}\ln\left[\lambda M(j)\right]$ (5)

where *M*(*j*) is the moment generating function of the dispersal kernel. The WALD model is integrated over terminal velocity and seasonal wind conditions to give the seasonal dispersal kernel (see also[3] for computational details):

(6)

where *p*(*F*) and *p*(*U*) are the probability density function of *F* and *U*, respectively, and *p*(*r*) is the WALD model. *k*(*r*) gives the radial dispersal kernel in a two-dimensional landscape. To estimate the rate of spread in one dimension *x*, we obtained the moment generating function for the dispersal kernel marginalized for *x* [8]. We estimated the empirical moment generating function by simulating a large number (*N* = 100,000) of random dispersal distances *r* using *k*(*r*) and random directions *α* using a uniform distribution (0-2*π*), and then calculated *x* = *r*cos(*α*). We used the medians of these runs to exclude potential extreme outliers arising from the integrated WALD model. The empirical moment generating function *M^E^*(*s*) is:

$M^{E}(w)=\frac{1}{N}\sum_{i=1}^{N} \exp(wx)$ (7)

where *w* is an auxiliary variable. This joint model then allowed us to project population spread (*c**) rates for all climate change scenarios.

*c**-LTRE Analyses

We used *c**-LTRE analyses to assess the contributions of changes in the vital rates and dispersal-related traits to changes in spread rates (*c**). We calculated the contributions of each altered parameters under climate change:

$\frac{\partial c*}{\partial\tau_{q}}=\sum\frac{m_{ij}(w*)\partial\rho_{1}\partial h_{ij}}{w*\rho_{1}\partial h_{ij}\partial\tau_{q}}$ (8)

Where τ_q_ is the qth vital rate, h_ij_ is the element at row i and column j of H, *w** is the auxiliary value corresponding to *c**, and ρ_1_ is the dominant eigenvalue of H. We calculated the sensitivity of *c** to plant height by introducing small perturbations [3]. We used the median of 1000 repeated calculations to avoid potential outliers.

**Supporting references**

1. Shea K, Kelly D (1998) Estimating biocontrol agent impact with matrix models: *Carduus nutans* in New Zealand. Ecological Applications 8: 824-832.

2. Shea K, Jongejans E, Skarpaas O, Kelly D, Sheppard AW (2010) Optimal management strategies to control local population growth or population spread may not be the same. Ecological Applications 20: 1148-1161.

3. Jongejans E, Shea K, Skarpaas O, Kelly D, Sheppard AW, et al. (2008) Dispersal and demography contributions to population spread of *Carduus nutans* in its native and invaded ranges. Journal of Ecology 96: 687-697.

4. Katul GG, Porporato A, Nathan R, Siqueira M, Soons MB, et al. (2005) Mechanistic analytical models for long-distance seed dispersal by wind. American Naturalist 166: 368-381.

5. Skarpaas O, Shea K (2007) Dispersal patterns, dispersal mechanisms, and invasion wave speeds for invasive thistles. American Naturalist 170: 421-430.

6. Evans M., Hastings N, Peacock B (1993) Statistical distributions, 2nd edition. John Wiley and Sons, New York.

7. Neubert MG, Caswell H (2000) Demography and dispersal: Calculation and sensitivity analysis of invasion speed for structured populations. Ecology 81: 1613-1628.

8. Lewis MA, Neubert MG, Caswell H, Clark JS, Shea K (2006) A guide to calculating discrete-time invasion rates from data. In: Marc WC, McMahon SM, Fukami T, editors. Conceptual ecology and invasion biology: reciprocal approaches to nature. Invading Nature - Springer Series in Invasion Ecology Volume 1. Springer, Dordrecht, The Netherlands. pp 169-192.

9. Lee JM, Hamrick JL (1983) Demography of two natural populations of musk thistle (*Carduus nutans*). Journal of Ecology 71: 923-936.
